# Efficacy and Safety of Dimethyl Fumarate in Patients with Moderate-to-Severe Plaque Psoriasis: DIMESKIN-2, a Multicentre Single-Arm Phase IIIb Study

**DOI:** 10.3390/jcm11164778

**Published:** 2022-08-16

**Authors:** Giovanni Pellacani, Laura Bigi, Aurora Parodi, Martina Burlando, Caterina Lanna, Elena Campione, Franco Rongioletti, Cristina Mugheddu, Giovanna Malara, Giovanna Moretti, Luca Stingeni, Katharina Hansel, Giuseppe Micali, Luigi Naldi, Federico Pirro, Ketty Peris

**Affiliations:** 1 Dermatology Unit, Department of Surgical, Medical, Dental and Morphological Sciences Related to Transplant, Oncology and Regenerative Medicine, University of Modena and Reggio Emilia, 41121 Modena, Italy; 2 Department of Clinical Internal, Anesthesiological and Cardiovascular Sciences, Dermatology Clinic, Sapienza University of Rome, 00185 Rome, Italy; 3 Section of Dermatology (DiSSal), University of Genoa, Ospedale-Policlinico San Martino IRCCS, 16132 Genova, Italy; 4 Dermatology Unit, University of Rome “Tor Vergata”, 00133 Rome, Italy; 5 Vita Salute University IRCSS San Raffaele Hospital, 20132 Milan, Italy; 6 Dermatology Clinic, University of Cagliari, 09124 Cagliari, Italy; 7 Grande Ospedale Metropolitano di Reggio Calabria, 89124 Reggio Calabria, Italy; 8 A.O. Papardo U.O.C. Dermatologia, 98158 Messina, Italy; 9 Dermatology Section, Head of Resident School of Dermatology and Venereology, Department of Medicine and Surgery, University of Perugia, 06132 Perugia, Italy; 10 Azienda Ospedaliero—Universitaria “Policlinico—Vittorio Emanuele” P.O. Gaspare Rodolico U.O.C. di Dermatologia Catania, 95123 Catania, Italy; 11 Azienda ULSS 8 Berica-Ospedale San Bortolo U.O. di Dermatologia, 36100 Vicenza, Italy; 12 Dermatology Unit, Catholic University of Rome, Fondazione Policlinico A. Gemelli, 00168 Rome, Italy

**Keywords:** dimethyl fumarate, psoriasis, PASI, BSA, PGA, quality of life, safety

## Abstract

This open-label multicentre trial evaluated the efficacy and safety of oral dimethyl fumarate (DMF) in patients with moderate-to-severe plaque psoriasis in real-life clinical practice over 52 weeks. Disease severity and improvement were assessed using the psoriasis area severity index (PASI), body surface area (BSA) affected, and Physician Global Assessment (PGA). Quality of life (QoL) was assessed using the Dermatology Life Quality Index (DLQI) questionnaire. The visual analogue scale (VAS) was used to quantify pruritus and measure treatment satisfaction. A total of 141 patients were included, being 66.7% male, aged 49.1 ± 14.7 years and with disease duration of 16 ± 12.1 years. After 52 weeks, mean PASI decreased from 15.9 ± 6.8 to 1.5 ± 2 and 87.7%, 56.9% and 24.6% of patients achieved PASI 75/90/100 response, respectively. BSA decreased from 26.5 ± 14.8% to 2.7 ± 3.5% at 52 weeks, and 81.5% of patients had a PGA 0-1. DLQI scores decreased from 9.4 ± 6.4 to 2.1 ± 3.3, and VAS of pruritus decreased from 53 ± 28.4 to 19.1 ± 26.2 at Week 52. VAS for treatment satisfaction was 79.4 ± 29.4 at Week 52. A total of 34.2% of patients had an AE leading to permanent discontinuation. These findings show that DMF can significantly improve indices of disease severity, pruritus and QoL, with high levels of patient satisfaction and similar safety profile to other fumarates.

## 1. Introduction

Plaque psoriasis is a chronic, inflammatory, immune-mediated skin disease affecting 2–3% of the population worldwide [1] and having significant impact on quality of life (QoL) [2,3].

Biological therapies such as tumour necrosis factor (TNF), interleukin (IL) 12/23 and IL-17 and IL-23 inhibitors have revolutionised the treatment of moderate-to-severe psoriasis allowing many patients to achieve complete remission [4,5,6]. However, many patients still go untreated or do not respond to biological therapies, or experience treatment-related toxicities [7,8,9]. Indeed, the outcome of systemic treatments varies from subject to subject and is often accompanied by significant side effects. For patients with moderate-to-severe psoriasis, the achievement of adequate long-term disease control with continued administration of safe and effective treatments is highly desirable [4,5]. Therefore, it is important to identify novel therapies as part of a systemic treatment that can offer a better risk/benefit ratio in terms of efficacy and tolerability, particularly in the long term [10].

Fumaric acid esters (FAEs) are a type of systemic treatment based on small molecules that have demonstrated a good tolerability and efficacy profile in patients with moderate to severe plaque psoriasis [10,11,12,13]. FAEs were approved as first-line treatment in Germany, where they currently represent one of the most common systemic treatments for plaque psoriasis [11,12,13]. In other European countries, no indication for use or guidelines are established yet [14]. FAEs consist of the combination of DMF and three monoethyl fumarate salts. FAEs have immunomodulatory, anti-proliferative, anti-inflammatory and apoptotic effects [11,12,13,15]. DMF is considered to be the only active ingredient in the mixture, since monoethyl fumarate salts alone have not been shown to have clinical efficacy [10,15].

The main clinical evidence evaluating the efficacy of DMF to date is based on the BRIDGE study, a double-blind, randomised, placebo-controlled trial, where DMF was evaluated after 16 weeks’ treatment using fumaric acid esters (FAEs; Fumaderm^®^; Biogen Idec GmbH, Munich, Germany) as an active comparator [10]. It was found that DMF was not inferior to Fumaderm^®^, showing a PASI75 score at 16 weeks of 37.5%, compared with 15.3% for placebo- and 40.3% of Fumaderm^®^-treated patients. In recent years, DMF has been evaluated in the real-life setting, but these studies are frequently limited by a small number of patients or a short follow-up period [16,17]. The aim of the present study was to evaluate the efficacy and safety of DMF in patients with moderate-to-severe plaque psoriasis in real-life clinical practice up to 1 year.

## 2. Materials and Methods

### 2.1. Patients

DIMESKIN-2 was a multicentre, open-label study to evaluate the efficacy and long-term tolerability of DMF (Skilarence^®^; Almirall Italia S.p.A., Milan, Italy) treatment in adults with moderate to severe chronic plaque psoriasis up to 52 weeks. This study was conducted by 36 investigators at 36 sites across Italy from May 2018 to November 2020.

Inclusion criteria were male or female patients aged ≥18 years with a diagnosis of chronic plaque psoriasis of at least 6 months prior to inclusion in the study. Moderate-to-severe plaque psoriasis was defined by at least one of the following criteria: PASI ≥ 10, BSA ≥ 10 and PASI ≥ 5 or DLQI ≥ 10 and PASI ≥ 5 [18]. All patients had good general health or a stable medical condition that did not interfere with the conduct of the clinical study, according to the assessment of the Investigator based on medical history, physical examination and results from laboratory tests. All patients were negative for hepatitis B, hepatitis C, HIV and tuberculosis. All patients had indication for systemic psoriasis treatment, a known history of at least 6 months of other previous topical and systemic treatments and a wash-out period of at least 4 half-lives (in the case of previous treatment with biological drugs). For females, a negative serum pregnancy test at the screening visit and willingness to use highly effective contraceptive methods was required. Exclusion criteria were a diagnosis of guttate, erythrodermic or pustular psoriasis, the presence of haematological abnormalities, history of malignancies (except non-melanoma skin cancers within the previous 5 years), significant gastrointestinal disturbances, known severe renal insufficiency (estimated glomerular filtration rate <30 mL/min), abnormal liver function, current infectious diseases, history of alcohol or drug abuse, known hypersensitivity to DMF components, known lactose intolerance, previous enrolment in this study or participation in other clinical pharmacology studies within the past 30 days. Other exclusion criteria (for female patients) included pregnancy status, planned pregnancy or lactation.

This study complies with the ethical standards of the 1975 Declaration of Helsinki, and the study (protocol number M-41008-42; DIMESKIN-2) was approved by the local Ethical Committee and registered under EudraCT identifier: 2017-003818-11). All patients provided written informed consent before inclusion in the study, in compliance with Italian Legislative Decree (L. 675/1996).

### 2.2. Study Design and Treatment

Patients underwent a baseline visit and visits during treatment on week 4, 8, 12, 24, 36, 48 and 52. For each visit, a time frame of 3 days before and after the scheduled date was allowed. Oral DMF treatment was administered as indicated in the summary of product information (SmPC) [19]. In the first week, patients took one 30 mg tablet of DMF once a day (in the evening), and in the second week, one 30 mg tablet of DMF twice a day (one tablet in the morning and one tablet in the evening). In the third week, patients received one 30 mg tablet of DMF three times a day (one tablet in the morning, one tablet at noon and one tablet in the evening). In the fourth week of treatment, the dosage was one 120 mg tablet of DMF once a day (in the evening). In subsequent weeks, the dose was increased by one 120 mg DMF tablet per week for the next 5 weeks (Week 5 to Week 9). From Week 10 through to Week 52, patients could take up to 2 120 mg DMF tablets three times a day, reaching a maximum of 720 mg DMF per day. However, it was not necessary to increase the dose before reaching the maximum dose of 720 mg/day if a 90% or greater improvement was observed in PASI (PASI90).

At any time and in case of significant intolerance based on their perception, patients restored the last tolerated dosage of DMF. Patients were also instructed to inform the Investigator immediately, including communication by telephone. The Investigator was responsible for taking the final decision on the restoration of the dosage or on the possibility of treatment interruption (temporarily or permanently). However, changes in the dosage could be necessary in case of alteration of the laboratory parameters: these changes were implemented by the Investigator based on the indications given in the SmPC of the drug or according to their own medical judgment. From Week 24, if a significant improvement in skin lesions was observed (defined as achieving a PASI75 or a PASI score of less than or equal to 3) and if the Investigator deemed it appropriate, the daily dose of DMF was gradually reduced—to the extent of one tablet less a day every two months—until the maintenance dose required by the patient’s condition was reached. From Week 24, the use of anti-H1 antihistamines, emollients without salicylic acid and emollients containing less than 10% urea was allowed.

### 2.3. Efficacy Measures

The primary objective of this study was to evaluate the efficacy of DMF in adult patients affected by moderate-to-severe chronic plaque psoriasis in terms of the proportion of patients achieving a ≥75% reduction from baseline of the Psoriasis Area and Severity Index (PASI) index after one year of treatment. Secondary objectives included the evaluation of the evolution of psoriasis at each visit over the 52 weeks using the PASI, Physician’s Global Assessment (PGA) and Body Surface Area (BSA). The PGA provides an evaluation of treatment efficacy by means of a score ranging from 0 (no sign of psoriasis) to 5 (severe psoriasis) [20].

### 2.4. Quality of Life Measures

Quality of life was assessed using the Dermatology Life Quality Index (DLQI) at each visit over the treatment period. The DLQI questionnaire is based on 10 questions and provides an evaluation of a patient’s QoL by means of a score ranging from 0 (no disease impact) to 30 (maximum disease impact) [21]. The higher the score, the more QoL is impaired.

Pruritus/itching and grade of satisfaction were evaluated at each visit by means of the visual analogic scale (VAS). Pruritus VAS is a visual method to assess the intensity of itching. The patient was asked to place a single mark along a 100 mm line; the left extremity represents the absence of itching (i.e., pruritus VAS equal to 0) and the right one unbearable itching (i.e., pruritus VAS equal to 100). VAS was also used to assess satisfaction of treatment over the 52 weeks, where a VAS of 0 represents complete lack of satisfaction and a VAS of 100 represents complete satisfaction.

### 2.5. Safety

The following safety parameters were monitored/collected during the study: treatment exposure and dosage, concomitant medications, adverse events, safety laboratory tests including haematology, clinical chemistry and urinalysis locally assessed at each participating site, vital signs, physical examination, and pregnancy test.

### 2.6. Statistical Analysis and Sample Size

Three hundred patients were planned to be enrolled in the present study. Defining as success the achievement of a ≥75% reduction from baseline in the PASI index at Week 52 (primary endpoint of the study), the sample size was determined in order to calculate the 95% confidence interval (CI), two-tailed, for the expected success rate of 50%. In order to obtain an accuracy of the estimate of the proportion of patients less than or equal to ±6.9%, 200 patients were to be enrolled in the present study. Considering a 35% expected dropout rate, the sample size was adjusted to 300 patients according to the Friedman formula: n’ = 100 × n/100 − x, where x is the expected dropout rate [22]. Data are presented as mean and standard deviation (SD) for continuous variables and number and % for frequencies. For the main variables of efficacy and safety, a descriptive analysis was carried out, and the 95% confidence intervals were calculated for means and proportions. Missing values of the primary and secondary efficacy endpoints, i.e., PASI75, PASI90, PASI100, PGA, BSA, DLQI, VAS for pruritus and level of satisfaction, were replaced using the multiple imputation (MI) method as the primary method and the Last Observation Carried Forward (LOCF) method for sensitivity analysis. The main efficacy endpoint was analysed in the Intention To Treat (ITT) population and in the Per Protocol (PP) population as a supportive analysis. Secondary efficacy endpoints were analysed in the ITT population. Mixed linear-effects models were used to compare changes in secondary efficacy variables over time. A *p*-value of <0.05 was considered statistically significant. Statistical analysis was performed using SAS^®^ (SAS Institute Inc., Cary, NC, USA) for Windows release 9.4 (64-bit) or later.

## 3. Results

### 3.1. Baseline Clinical Characteristics of Patients

In this multicentre single-arm phase IIIb study, a total of 141 patients with moderate-to-severe psoriasis were treated with DMF for a period of 1 year. Of the 141 patients who initiated treatment, 66 (46.8%) completed the study. Baseline characteristics of patients are presented in Table 1. The majority of patients were male (66.7%) with mean age of 49.1 ± 14.7 years and long history of psoriasis (mean disease duration of 16 ± 12.1 years). Approximately half of patients received treatment for psoriasis in the past 6 months (*n* = 75; 53.2%), and topical creams were the most frequent treatments (*n* = 55; 39%) followed by systemic treatment in 28 (19%) patients. Among the 75 patients with at least one prior psoriasis treatment, the mean time from the end of the last prior psoriasis treatment to the start of the study treatment was 71.2 ± 43.7 days. Abnormal physical examinations were reported in a small proportion of patients at baseline visit (*n* = 10; 7.2%).

### 3.2. PASI Response

**Figure 1:**
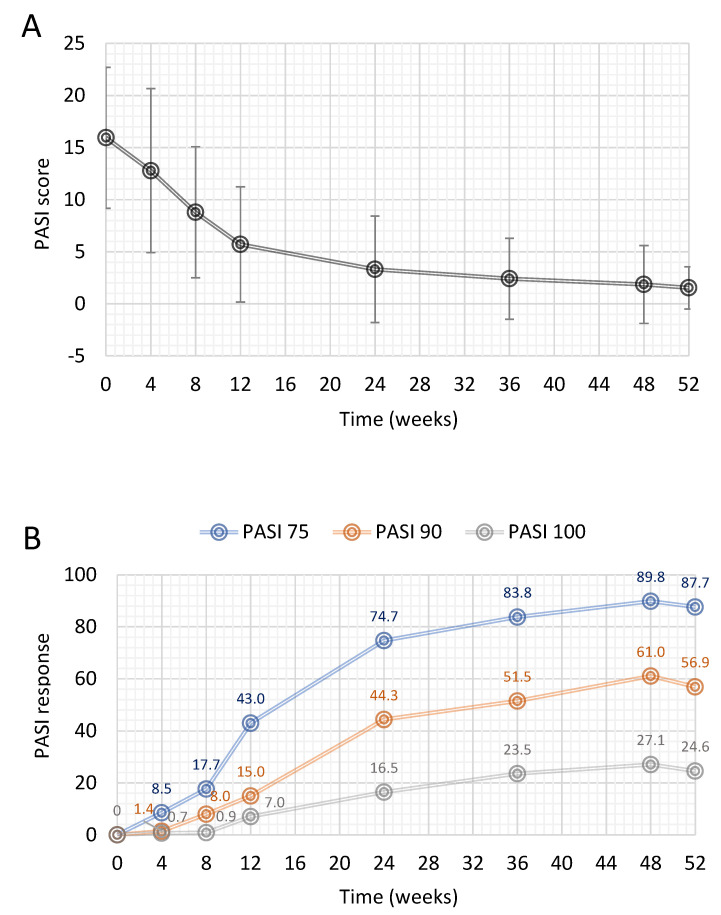
Effect of DMF in psoriatic patients on PASI score and achievement of PASI 75, 90 and 100 responses over 1 year. (**A**) Change in PASI over the treatment period. Data are presented as mean ± SD. (**B**) Proportion of responder patients achieving PASI75, PASI90 or PASI100 response at each visit using the available case analysis (AC) approach in the intention to treat population. PASI = Psoriasis Area and Severity Index. Each patient achieving a PASI reduction vs. baseline greater than or equal to a specific cut-off value (i.e., 75% for PASI75, 90% for PASI90 and 100% for PASI100) was considered as a ‘responder’.

Mean PASI total score decreased from 15.9 ± 6.8 at baseline to 1.5 ± 2. after 52 weeks, equating to an absolute change from baseline of −13.1 (95% CI: −14.4; −11.8) (Figure 1A). This decrease was statistically significant (*p* < 0.0001 for each visit) (Figure 1A). At 52 weeks, PASI 75, 90 and 100 responses were achieved in 87.7%, 56.9% and 24.6% of patients, respectively (Figure 1B). For the primary efficacy outcome (PASI 75 response), according to the MI approach, PASI75 at 52 weeks was 86.7% (95% CI: 78.7%; 94.6%) in the ITT population (*n* = 141). This result was also confirmed following the LOCF approach, showing a PASI75 at Week 52 of 81.1% (95% CI: 72.2%; 90%).

### 3.3. PGA and BSA

**Figure 2:**
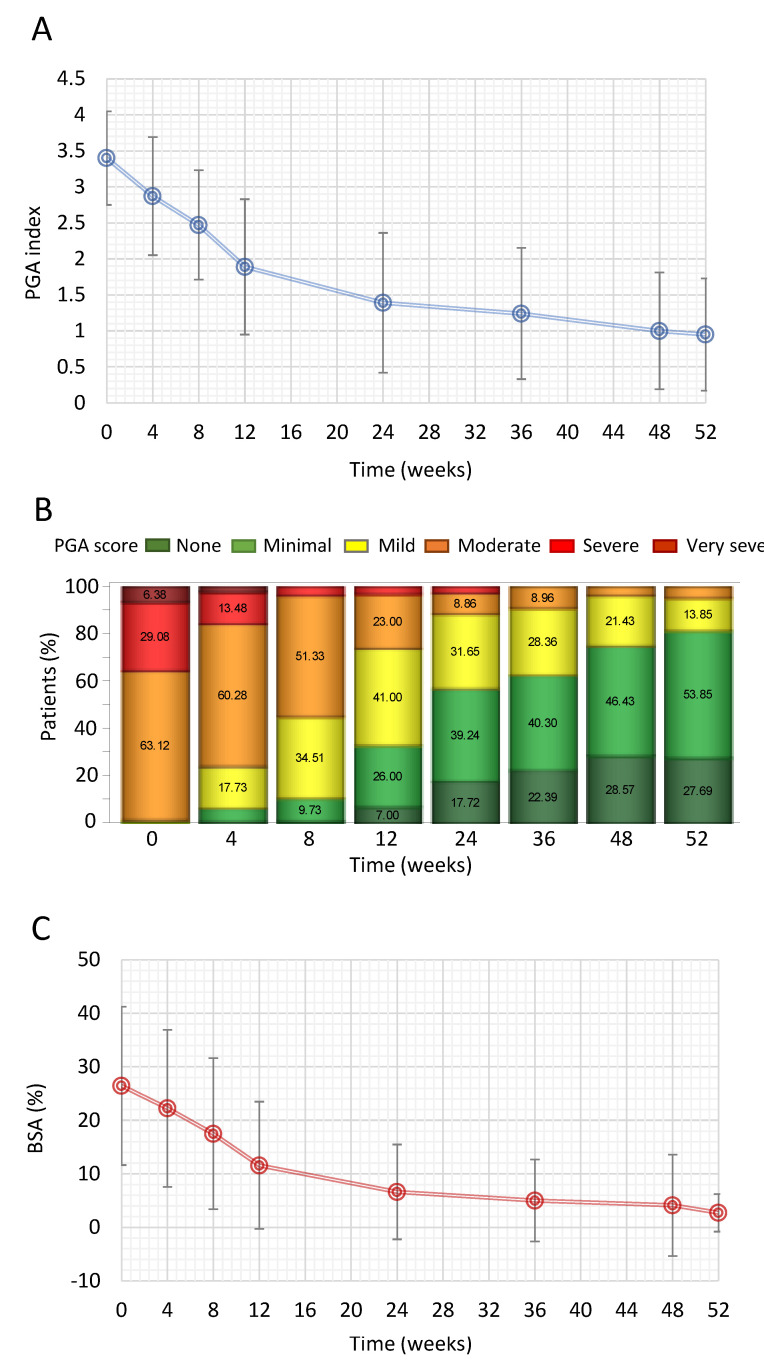
Effect of DMF in psoriatic patients on PGA index and BSA over 1 year. (**A**) Change in mean PGA index from baseline to 52 weeks. (**B**) Proportion of patients categorised as having ‘None’, ‘Minimal’, ‘Mild’, ‘Moderate’, ‘Severe’ and ‘Very severe’ disease according to PGA index. (**C**) Mean change in mean BSA % from baseline to 52 weeks. Data are presented as mean ± SD for PGA and BSA. BSA = body surface area, PGA = physician’s global assessment.

The mean PGA index decreased from 3.4 ± 0.65 at baseline to 0.95 ± 0.78 after 52 weeks, resulting in a mean absolute change of −2.3 ± 0.87 (Figure 2A). PGA index was also analysed in terms of categorical variable according to the class ‘None’, ‘Minimal’, ‘Mild’, ‘Moderate’, ‘Severe’ and ‘Very severe’, corresponding respectively to values 0, 1, 2, 3, 4 and 5. This analysis revealed that the proportions of patients in ‘Very severe’, ‘Severe’ and ‘Moderate’ categories decreased by visit in favour of an increase in the proportions of patients categorised with ‘None’, ‘Minimal’ and ‘Mild’ disease categories. At 52 weeks, the proportion of patients having ‘None’ or ‘Minimal’ disease was 81.5%, while 13.9% had ‘Mild’ disease and <5% of patients had ‘Moderate’ disease (Figure 2B). Mean BSA index decreased from 26.5 ± 14.8 at baseline to 2.7 ± 3.5 at Week 52, equating to a mean absolute change of −20.74 (95% CI: −23.63; −17.84) (Figure 2C). The observed decreases in PGA and BSA index were statistically significant (*p* < 0.0001) according to respective linear mixed effects models for repeated measures by visit.

### 3.4. Quality of Life Measures

**Figure 3:**
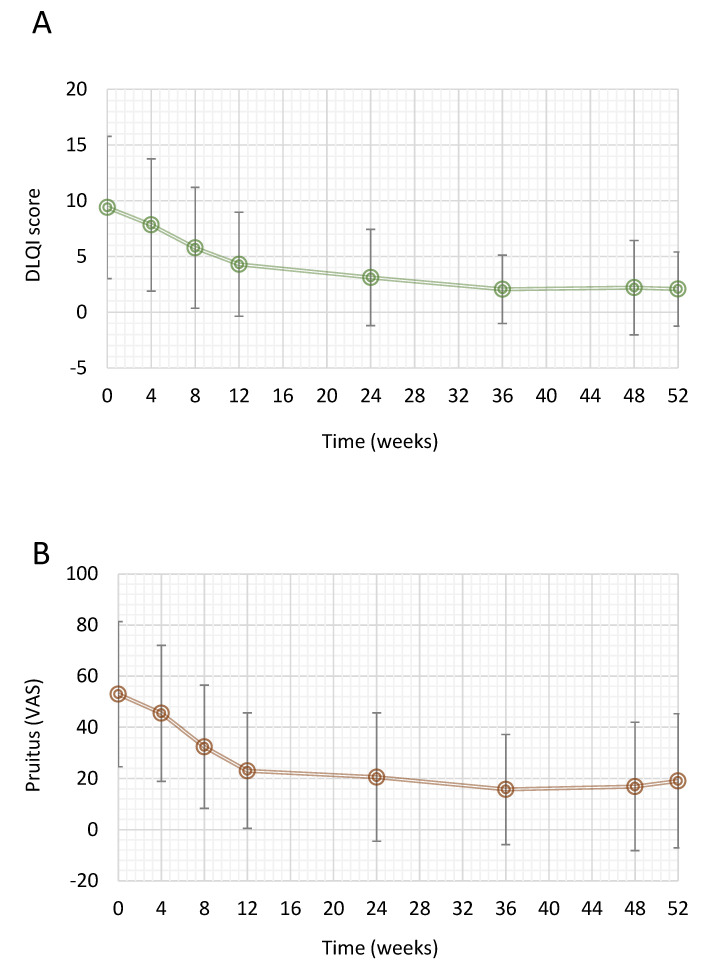
Effect of DMF in psoriatic patients on DLQI and pruritus VAS over 1 year. (**A**) Mean change in DLQI over the treatment period. (**B**) Mean change in pruritus VAS over the treatment period. Data are presented as mean ± SD. DLQI, Dermatology Life Quality Index; VAS, visual analogue scale.

Mean DQLI total score decreased from 9.4 ± 6.4 at baseline to 2.1 ± 3.3 after 52 weeks, resulting in a mean absolute change from baseline equal to −6.63 (95% CI: −7.91; −5.36) (Figure 3A). This decrease was statistically significant (*p* < 0.0001) according to the linear mixed effects model for repeated measures.

Mean pruritus VAS score decreased from 53 ± 28.4 at baseline to 19.1 ± 26.2 at Week 52, resulting in a mean absolute change from baseline equal to −31.3 (95% CI: −40.1; −22.4) (Figure 3B). This decrease was statistically significant (*p* < 0.0001) according to linear mixed effects model for repeated measures. We also assessed patient satisfaction with regard to study treatment evaluated by the satisfaction VAS score, and it was observed to be similar at Week 24 and Week 52, being 79.9 ± 23 and 79.4 ± 29.4, respectively.

### 3.5. Safety

Mean duration of exposure was 6.9 ± 5 months. All patients took 30 mg as the initial daily dosage, except for one patient who started the study treatment with 600 mg for three days. The patient interrupted treatment due to an AE and re-started with a daily dosage of 30 mg as per protocol. Mean daily dose of DMF increased over the first 9 weeks of treatment according to indication and as indicated in the SmPC [19] and was 350.6 ± 201.5 mg at Week 52. Treatment interruptions were observed in 58 patients (36.71%). The main reasons for temporary interruptions were ‘Decision of patient’ (102 out of 184; 55.4%), and the mean duration of each temporary interruption was 5.2 ± 11 days. At least one treatment-emergent adverse event (TEAE) was reported by 141 (89.2%) patients, and 127 (80.4%) patients reported at least one suspected drug-related TEAE. At least one severe TEAE was reported in 34 (21.5%) patients, and 7 patients (4.43%) reported at least one serious TEAE. Twenty-one patients (13.3%) had at least one TEAE that led to temporary interruption, while 54 patients (34.2%) had at least one TEAE that led to permanent treatment discontinuation. Only one patient (0.63%) had a fatal TEAE, which was assessed as unrelated (myocardial infarction) to the study treatment.

Four patients (2.53%) permanently discontinued treatment due to severe treatment-emergent serious adverse events: three patients (1.9%) due to lymphopenia and one patient (0.63%) due to generalised exfoliative dermatitis. The most commonly occurring AEs were related to ‘Gastrointestinal disorders’ reported by 59.5% of patients, ‘Skin and subcutaneous tissue disorders’ reported by 36.7% of patients, ‘Blood and lymphatic system disorders’ reported by 25.9% of patients, ‘Infections and infestations’ reported by 20.9% of patients and ‘Nervous system disorders’ reported by 17.19% of patients. The analysed haematology, biochemistry and urinalysis parameters were normal at each visit with the exception of white blood cells and absolute lymphocytes, which slightly decreased from screening to Week 52.

## 4. Discussion

This multicentre, single-arm, phase IIIb study investigated the efficacy and tolerability of DMF treatment (Skilarence^®^) in patients suffering from moderate-to-severe chronic plaque psoriasis.

Our findings show that in psoriatic patients treated with DMF for 1 year, PASI score decreased from 15.9 ± 6.8 to 1.5 ± 2 and 87.7%, and 56.9% and 24.6% of patients achieved PASI 75/90/100 response, respectively. BSA, PGA and DLQI as well as pruritus and treatment satisfaction all significantly improved over the 52 weeks.

The primary efficacy endpoint of this study was the proportion of patients achieving a 75% reduction in PASI (PASI75) at 52 weeks of treatment. According to the multiple imputation (MI) approach, the percentage of patients achieving PASI75 at 52 weeks was found to be 86.67% in the ITT population. Similar results were observed following the last observation carried forward (LOCF) approach, while the analysis of the PP population further corroborated these findings. The LOCF approach gave a PASI75 of 81.08%, which is confirmatory of the results obtained through the MI approach.

The main clinical evidence regarding the efficacy of DMF so far has been established in a double-blind, randomised, placebo-controlled trial (BRIDGE), where DMF was evaluated after 16 weeks’ treatment, using fumaric acid esters (FAEs; Fumaderm^®^) as an active comparator [10]. It was found that DMF was non-inferior to Fumaderm^®^, showing a PASI75 score at 16 weeks of 37.5%, compared with 15.3% for placebo- and 40.3% of Fumaderm^®^-treated patients. In the current study, a higher PASI75 value was reported at 52 weeks (87.7%), suggesting a sustained clinical efficacy for DMF in reducing psoriasis severity. Previous studies have shown similar results regarding short- and long-term efficacy of oral FAEs. One retrospective study demonstrated a PASI75 of 76% after 1 year of FAEs treatment [11]. In a Cochrane review of 2015, meta-analysis results showed a combined PASI50 response (percentage of patients achieving at least a 50% reduction in PASI score) of 64% with FAEs, compared with 14% with placebo at 12–16 weeks [23]. These findings are proof that DMF may be applied in patients with severe-to-moderate plaque psoriasis, suggesting for the treatment to be considered effective.

PASI index alone is not considered sufficient to evaluate psoriasis severity. The EMA recommends using two endpoints to assess efficacy, including a validated standardised global sore, such as the PGA score, in conjunction with PASI [24].

Both PGA and BSA scores significantly improved following DMF treatment. These results corroborate the findings of previous studies on DMF and FAEs treatment for moderate-to-severe psoriasis. Specifically, in the BRIDGE study, PGA scores of ‘clear’ or ‘almost clear’ were achieved by 33% and 37.4% of patients at 16 weeks of DMF or Fumaderm^®^ treatment, respectively, compared to 13% of placebo-treated patients, while BSA score was also shown to be significantly reduced at 8 and 16 weeks of DMF treatment, compared with placebo [10]. In another large prospective study, 76% of psoriasis patients were ‘markedly improved’ or ‘clear’ according to PGA scoring following one year of FAEs treatment [25]. Moreover, in a previous prospective study including 176 patients with moderate-to-severe psoriasis, a significant reduction in PGA score of 1.7 points was observed following a median DMF treatment of 28 weeks [26].

Health-related quality of life (HRQoL) is considerably affected by dermatological diseases, including psoriasis [27]. The DLQI is the most commonly used tool for assessing psoriasis impact on HRQoL [21]. Here, mean DLQI total score significantly decreased by visit, from 9.4 ± 6.4 at baseline to 2.1 ± 3.33 at Week 52, resulting in a mean absolute change from baseline of −6.63 (95% CI: −7.91; −5.36). In a post-hoc analysis of the BRIDGE study, DMF-treated patients reported a mean 5.9-point reduction in DLQI following 16 weeks of treatment [28]. In another observational study in patients treated with fumarates, an 8.9-point improvement in mean DLQI was observed after 12 months of treatment [12]. These results may be indicative of treatment success, given that a minimum clinically meaningful change in patients’ HRQoL has been demonstrated to correlate with a change of 5 points in the DLQI score [29,30]. Furthermore, a European expert consensus, as well as UK and European guidelines, define adequate response to psoriasis treatment as a 75% reduction in the PASI score or a 50% reduction in the PASI score and a 5-point reduction in DQLI from when treatment started [31,32,33].

A statistically significant decrease by visit in itching was also recorded based on the pruritus VAS scale (0–100), resulting in a mean absolute change from baseline equal to −31.27 (95% CI: −40.10; −22.44) after 52 weeks of treatment. Pruritus is considered one of the most bothersome symptoms in psoriasis, with a prevalence of 60–93% of patients [34]. This study is the first to our knowledge to evaluate the effect of DMF treatment on itching, showing a beneficial impact on symptom relief. The effects of DMF on pruritus improvement are comparable with other systemic psoriasis treatments. In a recent systematic review, meta-analysis of 13 studies showed that systemic treatments, including anti-IL-17, JAK inhibitors, adalimumab and apremilast, demonstrate a similar reduction in pruritus, ranging from −4.52 to −2.18 points, in a 0–10-point pruritus scale [35].

Importantly, treatment satisfaction was high, as reported by patients in a VAS (0–100) scale, being 79.9 ± 23 and 79.4 ± 29.39, at 24 and 52 weeks of treatment, respectively. High treatment satisfaction contributes to improved adherence to treatment, affecting treatment effectiveness in clinical practice [36].

The safety population of this study comprised 158 patients who took at least one dose of the study treatment. Overall, mean exposure time was 6.9 months. Given that most AEs first occur and are most frequent during the first few months of treatment, exposure time in this study is considered adequate to evaluate the safety profile of DMF [37]. Due to known side effects of FAEs, it is recommended that treatment is initiated with a low initial dose of 30 mg to improve tolerability, as instructed in the products SmPC [19]. Thus, all patients took 30 mg as initial daily dosage, except for one patient who started study treatment with 600 mg for three days, but interrupted treatment due to adverse events and re-started with a daily dosage of 30 mg as per protocol.

The mean daily dose increased as expected during the first weeks of treatment; however, it was lower than expected after Week 4. A mean daily dose of 350.6 ± 201.5 mg was reached at Week 52. In another multicentre study performed in Italy, Corazza and colleagues used a mean daily dose of 262.1 ± 190.9 mg and just over 40% of patients achieved a PASI 75 response by 26 weeks, suggesting that the lower dose may have limited the efficacy observed [17]. Interestingly, the proportion discontinuing the study due to AEs was 51.45%, which is higher than that observed in our study (34.2%). In our study, medication interruptions were observed in 36.71% of the patients, with the main reason being individual patient decision making.

Based on the observation of AEs, 89.24% of patients reported at least one treatment-emergent adverse event (TEAE), and 4.43% reported at least one serious TEAE. One serious TEAE (myocardial infarction) lead to one patient’s death, which was assessed as ‘unrelated’ to treatment. Most common adverse events were gastrointestinal disorders, reported mainly as cases of diarrhoea, nausea and abdominal pain, and other skin disorders, mostly erythema. Previous observations from other studies also point towards a similar safety profile regarding DMF and FAE treatment. Overall incidence of AEs in the BRIDGE study was demonstrated to be approximately 84%, both for DMF and Fumaderm^®^, while 23 serious TEAEs were reported in 3.2%, 2.8% and 3.6% of patients in the DMF, Fumaderm^®^, and placebo groups, respectively [10]. In the study of Lijnen et al. [26], 86% of patients reported one or more adverse events. In both studies, the most frequently reported TEAE was skin flushing and gastrointestinal complaints.

Permanent treatment interruption due to TEAEs was reported for 54 patients (34.2%), primarily due to gastrointestinal disorders (diarrhoea, nausea and abdominal pain) with similar rates observed by Pezzolo et al. recently in a real-world study [38]. High discontinuation rates have also been observed in the BRIDGE study, where 37% of DMF- and 38.5% of Fumaderm^®^-treated patients discontinued the study, with the main reason being occurrence of adverse events, observed in 23% and 24% of patients, respectively [10]. Gastrointestinal disorders are known and common side effects of FAEs, which occur mainly during the first weeks of treatment [23,39]. In general, treatment discontinuation may occur in up to 20% of patients due to gastrointestinal side effects of FAEs. However, dose adjustment is often sufficient to decrease their severity and ensure treatment compliance [33].

Leukopenia and lymphopenia may occur under treatment with FAEs and are reported as ‘very common’ side effects in the study product’s SmPC [40]. In this study, ‘Blood and lymphatic system disorders’ were reported in 25.95% of patients. Lymphopenia was the most common disorder, as reported in 23.4% of patients. Severe lymphopenia, however, was only reported in three (1.9%) patients. In the BRIDGE trial, 10% of patients in the DMF and in 10.6% in the Fumaderm^®^ group reported lymphopenia [10]. After long-term use of FAEs, lymphopenia has been reported in up to 41% and leukopenia in 12% of patients following 24 months treatment [25]. FAE-induced lymphopenia is not considered to lead to significant immunosuppression considering that lymphocyte counts are closely monitored, while only severe cases of prolonged lymphopenia have been linked to occurrence of opportunistic infections, such as progressive multifocal leukoencephalopathy [41]. Following FAEs treatment, lymphopenia is commonly mild, primarily experienced during the dosage increase phase, while it can be mostly managed with dose adjustments [33].

Here, laboratory data evaluation revealed that white blood cell and lymphocyte count slightly decreased from baseline visit to Week 52. The European S3 guidelines recommend measuring full blood count, liver enzymes, serum creatinine, urine sediment, and performing a pregnancy test before starting FAEs and every four weeks during treatment. If leucocytes drop below 3000/μL and lymphocytes below 500/μL, the dose must be reduced, or the treatment should be discontinued [32]. For Skilarence^®^, the European expert consensus recommends monitoring visits to be scheduled every 3 months and a complete blood count with differential to be performed [33].

Given that a high proportion of psoriasis patients (particularly those with moderate-severe disease) are burdened with co-morbidities as well as psoriasis lesions in difficult-to-treat areas (e.g., scalp, nails, genitals etc.), recent evidence suggests that DMF can also be effective in these cases [42,43], allowing dermatologists to offer this treatment in these difficult-to-treat patients.

## 5. Study Limitations

There were some limitations in this study that should be mentioned. The planned number of participants in the study was not reached and discontinuation rate was high, as out of the 165 patients enrolled, 99 (60%) discontinued the study. Of these, 54% discontinued due to AEs. Due to the high discontinuation rate, the LOCF approach may have ameliorated the reported treatment effect. Additionally, the MI approach implemented the Missing At Random (MAR) assumption in order to adjust the analysis with regard to the missing data. However, the primary challenge was that the MAR assumption cannot be evaluated since there is no knowledge of missing data, therefore it was impossible to make comparisons of observed and missing data to check for any systematic differences. Although similar results were observed for the primary efficacy measure (PASI 75 response) for the available case, LOCF and MI approaches used, the non-responder imputation approach may have produced more conservative results [44,45].

Current treatment guidelines on psoriasis include the use of biologics that have demonstrated high efficacy for patients with moderate-to-severe psoriasis. Biologic agents for psoriasis treatment include tumour necrosis factor α (TNF-α) inhibitors, interleukin (IL)-23 inhibitors, IL-12/23 inhibitors, or IL-17 inhibitors [46]. It is recognised that the presence of comorbid diseases in patients with psoriasis frequently dictates the type of treatment [47,48] offered, and this is particularly relevant in the case for fumarates [17,43]. The administration of biologics has been associated with side effects such as serious infections, cardiovascular events and cancers, mainly with first-generation biologic drugs [9]. Following the study’s findings, it can be concluded that DMF may be advantageous in treating moderate-to-severe psoriasis, and in terms of safety profile, in comparison to biologic treatments, DMF is not associated with increased risk of infections or malignancies [33]. Moreover, drug−drug interactions with regard to co-administered FAEs have yet to be noted, as with other systemic treatments [25].

## 6. Conclusions

This study confirmed the efficacy and safety of DMF for the treatment of moderate-to-severe psoriasis in the Italian population over a period of 52 weeks. As the efficacy of FAEs has been shown to increase over several months of treatment [25], the longer study duration employed here is paramount in assessing maximum efficacy. Thus, given the limited clinical data with DMF beyond 16 weeks, this study further contributes to the establishment of a long-term efficacy profile of Skilarence^®^. In addition, the long-term safety profile of DMF in this study was demonstrated to be similar to the previously described safety profile of Skliarence^®^ and FAEs. Due to known gastrointestinal side effects and the effect of FAEs on white blood cells, caution should be applied in individualised dose titration, lymphocyte count should be monitored, and patient expectations should be managed in order to ensure treatment compliance with minimal side effects. 

## Figures and Tables

**Table 1:** Baseline clinical characteristics of psoriasis patients.

Clinical Characteristic	*n* = 141
*General*	
Male gender, *n* (%)	94 (66.7)
Age (years)	49.1 ± 14.7
BMI (kg/m^2^)	26.6 ± 5.2
Current cigarette smoker, *n* (%)	60 (43.8)
*Disease characteristics*	
Disease duration	16 ± 12.1
PASI at baseline	15.9 ± 6.8
BSA affected (%)	26.5 ± 14.8
PGA index *	3.4 ± 0.65
Moderate disease	89 (63.1)
Severe disease	41 (29.1)
Very severe disease	9 (6.4)
DLQI score	9.4 ± 6.4
Pruritus (VAS)	52 ± 28.4
*Previous treatment n*, (%) *	75 (53.2)
Topical	55 (39)
Antipsoriatics	35 (24.8)
Corticosteroids	19 (13.5)
Emollients/protectives	1 (0.71)
Systemic	28 (19.9)
Antipsoriatics	10 (7.1)
Corticosteroids	2 (1.4)
Immunosuppressants	14 (9.9)
Phototherapy	2 (1.4)

BMI, body mass index; DLQI, Dermatology Life Quality Index; HBV/HCV, hepatitis B/C virus; PGA, physician’s global assessment; PASI, Psoriasis Area and Severity Index; TB, tuberculosis; VAS, visual analogue scale. Data presented as mean ± standard deviation or number and %. * Last psoriasis treatment within the past 6 months prior to starting the study.

## Data Availability

Not applicable.
